# Spatial Pattern of Technological Innovation in the Yangtze River Delta Region and Its Impact on Water Pollution

**DOI:** 10.3390/ijerph19127437

**Published:** 2022-06-17

**Authors:** Jianwei Zhang, Heng Li, Guoxin Jiao, Jiayi Wang, Jingjing Li, Mengzhen Li, Haining Jiang

**Affiliations:** 1 School of Resources, Environment and Tourism, Anyang Normal University, Anyang 455000, China; 01498@aynu.edu.cn (J.Z.); jgx0808x@126.com (G.J.); wangjiayizhuzhi@163.com (J.W.); lijingjing961@163.com (J.L.); lmz05608@163.com (M.L.); 2 School of Economics and Management, Huainan Normal University, Huainan 232038, China; 3 College of Geography and Environmental Sciences, Zhejiang Normal University, Jinhua 321004, China; jhn841263@zjnu.cn

**Keywords:** technological innovation, water pollution, spatial pattern, gray relational analysis, YRD

## Abstract

The impact of technological innovation on water pollution is an important parameter to determine and monitor while promoting and furthering a region’s economic development. Here, exploratory spatial data analysis was used to analyze: the spatial patterns of technological innovation and water pollution in the Yangtze River, the changes in technical innovation and the resulting changes in water pollution, and the impact of technological innovation on water pollution. The following major inferences were drawn from the obtained results: (1) The spatial pattern of innovation input has a single-center structure that tends to spread. The patent innovation output has evolved, from a single spatial pattern with Shanghai as the core to a diffusion structure with three cores-Hangzhou, Shanghai, and Nanjing. (2) The aggregation mode of water pollution has evolved from the original “Z” mode to a new mode of core agglomeration, and water pollution is constantly being reduced. (3) The trends of change in patent innovation output and innovation input are roughly the same, while the trends of both and that of water pollution are contrary to each other. (4) The correlations between innovation input, patented innovation output, and water pollution are relatively low. From the perspective of spatial distribution, the number of cities with medium and high levels of gray correlation with water pollution is the same.

## 1. Introduction

The extensive growth of China’s economy has increasingly intensified ecological imbalance and environmental pollution, particularly following its reform, opening up, and rapid growth. In response to this, the report by the 19th National Congress of the Communist Party of China stipulated the implementation of the most stringent ecological and environmental protection system in recent years. Environmental pollution control has entered a critical period, and various governments have formulated stringent environmental protection policies; however, this has affected economic development [1,2,3]. Technological innovation plays an important role in the trade-off and coordination of the extent and quality of economic growth, and outstanding economic development is dependent on the implementation of innovation-driven strategy [4,5,6,7,8]. Under the economic construction of the “new normal”, an efficient growth model driven by innovation as the core is the optimal choice for the achievement of both economic growth and environmental protection. Therefore, the relationship between environmental pollution and innovation ability has increasingly drawn attention.

The impact of technological innovation on environmental pollution is currently controversial. The first view suggests that technological innovation can significantly reduce environmental pollution as constant research and development can transform enterprises [9,10,11,12]. This can occur at the level of inter-provincial, municipal, or pollution-intensive industries [13,14,15,16,17,18]. Technological innovation can significantly reduce environmental pollution and offset the cost effect of pollution control through long-term technical effects. Industrial agglomeration can reduce pollution primarily because enterprises in industrial clusters use a greater number of environmental protection technologies for production [19,20,21,22,23,24,25,26]. Liu (2018) found that technological innovation could not only reduce haze pollution, but it could also indirectly reduce haze concentrations in neighboring provinces through knowledge spillover effects [27]. The second view suggests that technological innovation has a significant positive impact on environmental pollution emissions; this view is based on industrial industry and can be attributed to the fact that technological innovations focus on profitability in the industrial production process and ignore technological innovation for environmental protection; for example, there is a lack of green patents in the industry [28,29,30,31,32,33,34,35]. Green innovation can significantly improve environmental sustainability, but only under environmental regulation policy; likewise, technological innovation will promote the mitigation of environmental pollution [36,37,38,39,40,41]. The third perspective is based on studies of heavily polluting industrial enterprises in Shanghai and Shenzhen, and the findings revealed that technological innovation has no effect on the expanding transformation enterprises [42,43,44,45]. Lanoie [46] studied the causality of environmental policy, research and development and the environment, and business performance in seven member countries of the Organization for Economic Co-operation and Development; they found that investment in environmental research and development had no significant effect on environmental performance. The fourth view is that technological innovation has environmentally friendly characteristics up to a threshold value. In other words, when the level of technological innovation is low, environmental pollution cannot be reduced. The fifth perspective is that the role of technological innovation and industrial structure upgrading in eastern, central, and western China shows obvious regional differences [47,48,49,50,51,52,53,54,55]. Technological innovation in all cities negatively impacts environmental pollution, and the impact degree shows a gradually increasing spatial differentiation pattern from east to west. The sixth perspective is that innovative agglomeration can reduce haze pollution through the scale effect, but the effect of the innovative agglomeration spatial distribution mode on haze pollution is heterogeneous [56,57,58,59,60].

Generally, local and international studies on environmental pollution and technological innovation mainly conduct quantitative analysis with respect to time, and spatially, the role of technological innovation in environmental pollution is rarely examined. Owing to the differences in research objects, time period, index selection, and methods, the impact of technological innovation on environmental pollution is extensively debated. There is limited literature on water pollution and technological innovation alone; hence, the relationship between the two urgently needs to be examined from the spatial perspective. As one of the regions with robust innovation capacity in China, the innovation capacity of the Yangtze River Delta (YRD) region has been improved rapidly, and the environmental pollution has also been significantly mitigated. The impact of technological innovation on water pollution and the spatial effect of different types of innovation affecting water pollution urgently need to be addressed. The exploratory spatial data analysis (ESDA) method and center of gravity analysis can well reveal the relationship between technological innovation and water pollution. Thus, ESDA was used to analyze the spatial pattern of technological innovation and water pollution in the YRD, while the center of gravity analysis method was used to analyze the change in the center of gravity between technological innovation and water pollution. The gray correlation model was used to analyze the impact of technological innovation on water pollution in order to provide certain decision-making references and theoretical support for the scientific formulation of policies such as those improving technological innovation ability and ones that improve the environment in the YRD region and promote the high-quality development of the regional economy.

## 2. Methods and Data

### 2.1. Overview of the Study Area

**Figure 1:**
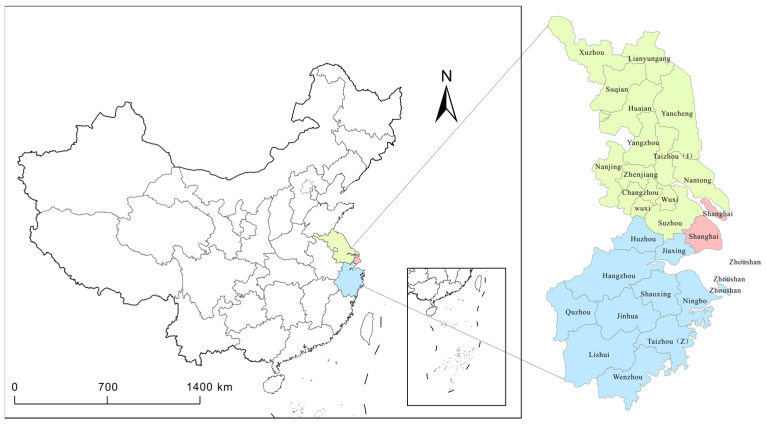
Overview map of the Yangtze River Delta.

With social and economic development, the planning scope of the YRD region will witness further changes. The core areas of the study are the provinces of Jiangsu, Zhejiang, and Shanghai. Jiangsu Province comprises 13 cities: Nanjing, Wuxi, Xuzhou, Changzhou, Suzhou, Nantong, Huaian, Yancheng, Yangzhou, Zhenjiang, Taizhou, Suqian, and Lianyungang; Zhejiang Province comprises 11 prefecture-level cities: Hangzhou, Ningbo, Wenzhou, Jiaxing, Huzhou, Shaoxing, Jinhua, Quzhou, Zhoushan, Taizhou, and Lishui; Shanghai Province covers an administrative area of approximately 217,900 km^2^. In 2019, the total number of patents granted in this region reached 1,076,994, which significantly exceeded those of other regions in China (Figure 1). The study area is a region with the highest innovation capacities in China and is a pioneer in and a model of national ecological environmental planning and management. At present, all cities in the YRD are included in the scope of air quality monitoring, with the total number of air quality monitoring stations being 162. However, energy consumption is high owing to the relatively developed industries, and environmental pollution continues to be a problem.

### 2.2. Data Sources and Processing

The data in this study are from the China Urban Statistical Yearbook from 2005 to 2020. For some provinces, the data for some of the years were missing. Therefore, based on the existing data, piecewise interpolation or average values before and after years were adopted to supplement and improve the assignment. The data were standardized, and the inverse index formula of water pollution data was processed to eliminate the dimensional influence.

Among them, innovation input is represented by scientific and technological expenditure, innovation output is represented by the number of patent authorizations, and water pollution is represented by industrial wastewater discharge. For the independent variables involved in the gray correlation method, this study assumed that innovation input and patent innovation output exert a certain impact on water pollution, represented by scientific and technological expenditure and the number of patent authorizations, respectively [61,62,63,64,65,66]. The proportion of secondary industry in GDP, green coverage rate of built-up areas, comprehensive utilization rate of general industrial solid waste, total population at the end of the year, and the actual amount of foreign capital used in the year were used to characterize the industrial structure, urban greening, environmental governance, population size, and foreign direct investment (FDI) factors [67,68,69,70,71,72] as well as the number of mobile phone users at the end of the year, while postal service revenue was used to characterize the innovation base.

### 2.3. Main Research Methods

#### 2.3.1. Analysis of Gravity Center Coupling

Coupling indicates the phenomenon of interaction between two or more systems, and the coupling degree reflects the degree of coordination between systems. In this study, the spatial distribution and coupling change characteristics of the technological innovation and water pollution centers are studied from static and dynamic directions, respectively, using spatial overlap and variation consistency. Among them, innovation input is represented by scientific and technological expenditure, innovation output is represented by the number of patent authorizations, and water pollution is represented by industrial wastewater discharge [73,74,75,76]. The innovation center of gravity includes the centers of innovation input and innovation output.

(1) Spatial overlap

Spatial overlap is usually represented by spatial distance (S). $S_{1}$ is $d_{G_{E}G_{P}},$ the spatial overlap between innovation input and water pollution.$\;S_{2}$ is $d_{G_{F}G_{P}},$ the spatial overlap of innovation output and water pollution. The smaller the spatial distance between the center of technological innovation and the center of water pollution, the higher the spatial overlap and the stronger the coupling, and vice versa [77,78,79]. The innovation gravity center includes the innovation input gravity center and the innovation output gravity center, which are represented by (*x_E_*,*y_E_*) and (*x_F_*,*y_F_*), respectively, while the coordinates of the water pollution gravity center are represented by (*x_P_*,*y_P_*). The calculation formula is as follows:

(1) $$S_{1}=d_{G_{E}G_{P}}=\sqrt{{\left(x_{E}-x_{P}\right)}^{2}+{\left(y_{E}-y_{P}\right)}^{2}}$$

(2) $$S_{2}=d_{G_{F}G_{P}}=\sqrt{{\left(x_{F}-x_{P}\right)}^{2}+{\left(y_{F}-y_{P}\right)}^{2}}$$

(2) Consistency of change

Variation consistency refers to the vector angle *θ* formed by the displacement of the two centers of gravity at the last time point. Because the vector angle is located within the range of 0–180°, the cosine value of *C* is used to measure the consistency of the change direction of the two barycenters. The larger the value of *C* is, the smaller *θ* is and the more consistent the change direction is. When *C* = 1, *θ* = 0 and the directions of change of the two centers of gravity are consistent. Similarly, when *C* = −1, the direction of change is completely the opposite. Δ*x_E_*, Δ*x_F_*, and Δ*x_P_* represent the longitudinal changes of the innovation input center of gravity, the innovation output center of gravity, and the water pollution center of gravity, respectively, relative to the previous time point. Δ*y_E_*, Δ*y_F_*, and Δ*y_P_* represent the latitudinal changes of the innovation input, innovation output, and water pollution, respectively. *C*1 represents the cosine of the center of gravity of innovation input and the center of gravity of water pollution. *C*2 represents the cosine of the center of gravity of innovation output and the center of gravity of water pollution.

C1=cosθ

(3) $$=\frac{\left(\Delta x_{E}{}^{2}+\Delta y_{E}{}^{2}\right)+\left(\Delta x_{P}{}^{2}+\Delta y_{P}{}^{2}\right)-\left[{\left(\Delta x_{E}-\Delta y_{E}\right)}^{2}+{\left(\Delta x_{P}-\Delta y_{P}\right)}^{2}\right]}{2\sqrt{\left(\Delta x_{E}{}^{2}+\Delta y_{E}{}^{2}\right)\left(\Delta x_{P}{}^{2}+\Delta y_{P}{}^{2}\right)}}=\frac{\Delta x_{E}\Delta x_{P}+\Delta y_{E}\Delta y_{P}}{\sqrt{\left(\Delta x_{E}{}^{2}+\Delta y_{E}{}^{2}\right)\left(\Delta x_{P}{}^{2}+\Delta y_{P}{}^{2}\right)}}$$

C2=cosθ

(4) $$=\frac{\left(\Delta x_{F}{}^{2}+\Delta y_{F}{}^{2}\right)+\left(\Delta x_{P}{}^{2}+\Delta y_{P}{}^{2}\right)-\left[{\left(\Delta x_{F}-\Delta y_{F}\right)}^{2}+{\left(\Delta x_{P}-\Delta y_{P}\right)}^{2}\right]}{2\sqrt{\left(\Delta x_{F}{}^{2}+\Delta y_{F}{}^{2}\right)\left(\Delta x_{P}{}^{2}+\Delta y_{P}{}^{2}\right)}}=\frac{\Delta x_{F}\Delta x_{P}{}^{2}+\Delta y_{F}\Delta y_{P}}{\sqrt{\left(\Delta x_{F}{}^{2}+\Delta y_{F}{}^{2}\right)\left(\Delta x_{P}{}^{2}+\Delta y_{P}{}^{2}\right)}}$$

#### 2.3.2. Gray Correlation Analysis

The gray correlation degree represents the degree of correlation between two factors. In theory, it uses an unclear sample information and an uncertain gray system as research objects to compute the comparative sequence distance between each point and to determine the differences and similarities in the sequence, as well as how they are related to the main influencing factors [80,81,82,83,84,85]. The correlation degree of each influencing factor is determined. The higher the correlation degree, the greater the impact of this influencing factor index on environmental pollution [84,86,87,88,89,90]. The gray correlation analysis method can only be performed in accordance with the development trend and does not require a certain probability distribution law and statistical test. It makes up for the shortcomings of statistical analysis and involves relatively lesser calculation; therefore, it has a wide range of application values [91,92,93]. This method involves nine independent variables and one dependent variable. The dependent variable is water pollution, represented by industrial wastewater discharge. This study assumes that innovation input and patent innovation output have a certain impact on water pollution, which are represented by scientific and technological expenditure and the number of patent grants, respectively [94,95,96,97,98,99]. In addition, according to relevant research, water pollution is also affected by factors such as industrial structure, urban greening, environmental governance, population size, FDI factors, and innovation foundations. These factors are represented by the proportion of the secondary industry in GDP, the green coverage rate of built-up areas, the comprehensive utilization rate of general industrial solid waste, the total population at the end of the year, the amount of foreign capital actually used in the year, the number of mobile phone users at the end of the year, and the income of postal services [100,101,102,103,104,105,106,107]. The specific calculation process is as follows:

① The reference and comparison sequences are determined. The reference series is an appropriate comparison standard, and other reference values can be selected for evaluation. The comprehensive water pollution index is regarded as the reference series $\;x_{0}$ and the influencing factor index as the comparison series $\;x_{i}$.

② The original data of the selected reference series and comparison series are standardized to make them dimensionless, and the mean value method is specifically used to improve the accuracy of the results. $\;x_{i}\left(k\right)$ represents the KTH sample of the ITH indicator, and the specific steps are as follows:

(5) $$x_{i}^{\prime }\left(k\right)=\frac{x_{i}\left(k\right)}{\frac{1}{n}\sum_{k=1}^{n}x_{i}\left(k\right)\;}\;i=0,1,\cdots ,n;\;k=0,1,\cdots ,m$$

③ The absolute difference sequence is calculated. The absolute difference between the comparison sequence and its corresponding reference sequence is calculated to yield the difference sequence and to determine the minimum value: $\underset{i=1}{\min}\;n\;\underset{k=1}{\min}\;m\;\left|x_{0}\left(k\right)-x_{i}\left(k\right)\right|$, and the maximum value: $\underset{i=1}{\max}\;n\;\underset{k=1}{\max}\;m\;\left|x_{0}\left(k\right)-x_{i}\left(k\right)\right|$.

④ The correlation coefficient $\delta_{i}\left(k\right)$ is calculated. The difference value is substituted into several formulas, and the correlation coefficient between the comparison sequence and the reference sequence is calculated. Here, ρ is the resolution coefficient, and the value interval of  ρ  is (0, 1), which is usually taken as 0.5.

(6) $$\delta_{i}\left(k\right)=\frac{\underset{i}{\min}\;\underset{k}{\min}\left|x_{0}\left(k\right)-x_{i}\left(k\right)\right|+\rho \;\underset{i}{\max}\;\underset{k}{\max}\left|x_{0}\left(k\right)-x_{i}\left(k\right)\right|}{\left|x_{0}\left(k\right)-x_{i}\left(k\right)\right|+\rho \;\underset{i}{\max}\;\underset{k}{\max}\left|x_{0}\left(k\right)-x_{i}\left(k\right)\right|}$$

⑤ The correlation degree $\;\gamma_{i}$ is measured. The average value of the correlation coefficient column of each index and the corresponding element of the reference sequence are calculated to indicate the correlation degree between the evaluation object and the reference sequence; ranking is also performed.

(7) $$\gamma_{i}=\frac{1}{m}\overset{n}{\underset{k=1}{\sum }}\delta_{i}\left(k\right)$$

(3) Exploratory spatial data analysis

The hotspot analysis method in ESDA was applied to analyze the agglomeration of technological innovation [81,82,83]. To reveal the contribution of each city to the global autocorrelation, Getisord $G_{i}$* was used to analyze the spatial clustering of high or low-value elements. The expression is as follows:

(8) $$G_{i}^{*}=\frac{\sum_{i=1}^{n}w_{ij}p_{i}}{\sum_{i=1}^{n}p_{i}}$$

The standardized statistic for the test is as follows:

(9) $$Z\left(G_{i}^{*}\right)=\frac{G_{i}^{*}-E\left(G_{i}^{*}\right)}{\sqrt{Var\left(G_{i}^{*}\right)}}$$

In Formulas (8) and (9), *E*(G_i_*) and *Var*(G_i_*) represent the mathematical expectation and the coefficient of variation of G_i_*, respectively, Wij is the spatial weight matrix based on Rook, and Pi is the observed value of city i. If *Z*(G_i_*) is positive and significant, it indicates that city *i* is a high-value area for technological innovation agglomeration and belongs to a hotspot (high-value space agglomeration). In contrast, if *Z*(G_i_*) is negative and significant, the city belongs to a cold spot area (low-value space agglomeration).

## 3. Spatial Pattern Analysis of Technological Innovation and Water Pollution in YRD

### 3.1. Spatial Pattern of Technological Innovation in YRD

**Figure 2:**
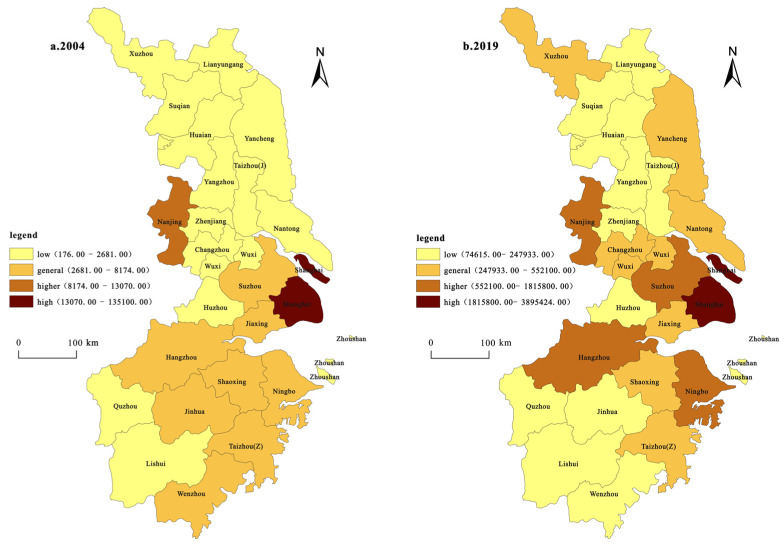
Spatial pattern change based on innovation input.

**Figure 3:**
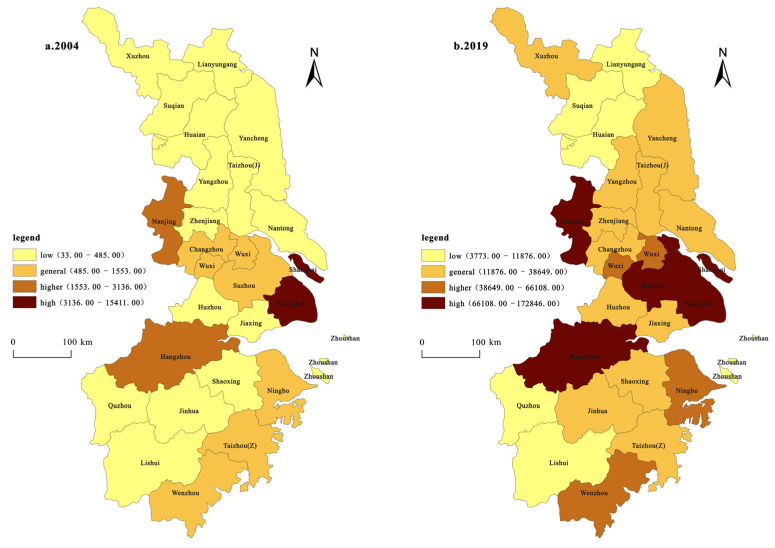
Spatial pattern change based on innovation output.

**Figure 4:**
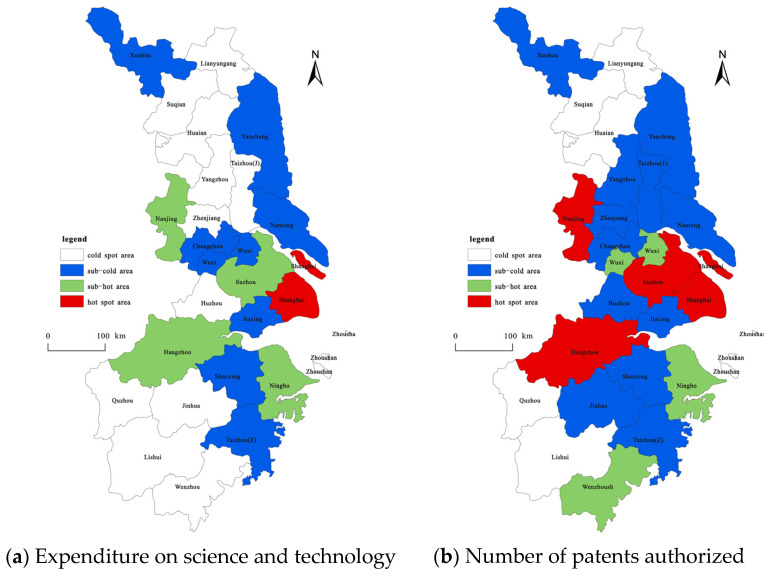
Spatial growth pattern of innovation inputs and outputs: 2004–2019.

(1) Spatial patterns based on innovation inputs (Figure 2). The innovation input is represented by scientific and technological expenditure, and the classification and mapping were carried out using the classification method of natural breaking points in the ArcGIS software. The spatial pattern of innovation investment in the YRD has a prominent single-center structure and exhibits a trend of diffusion. In 2004, Shanghai was the only city with a “high” investment in innovation, accounting for 62.9% of the total investment in the YRD. Nanjing city had the highest investment. Suzhou, Jiaxing, Hangzhou, Jinhua, Shaoxing, Ningbo, Taizhou, and Wenzhou were categorized as “general”, while the rest were categorized as “low”. In 2019, only Shanghai was categorized as “high”, while Suzhou, Hangzhou, and Taizhou were raised in rank to the “high” category. Jinhua and Wenzhou were categorized under “general”, Wuxi, Changzhou, Xuzhou, Yancheng, and Nantong were raised in rank to the “general” category, and the other cities remained unchanged.

(2) Spatial patterns based on innovation output (Figure 3). The innovation output is represented by the number of patent authorizations, and the classification and mapping were carried out using the natural breaking point classification method in the ArcGIS software. The spatial distribution of the innovation input was found to be moderately consistent with that of the innovation output. In 2004, Shanghai was the only city with a “high” innovation output, while Nanjing and Hangzhou were the two cities with high innovation output. Six cities were categorized as “general”: Changzhou, Suzhou, Wuxi, Ningbo, Taizhou, and Wenzhou, accounting for approximately 1/4, and the rest were categorized as “low”. In 2019, four cities were categorized as “high”, with Shanghai remaining in the “high” category, while Nanjing, Suzhou, and Hangzhou cities were raised in rank to the “high” category. The number of cities in the “high” category increased to three. The cities of Wuxi, Ningbo, and Wenzhou increased from 6 to 12, accounting for almost 1/2. Changzhou and Taizhou were always categorized as “general”. Xuzhou, Nantong, Yancheng, Yangzhou, Zhenjiang, Taizhou, Jiaxing, Huzhou, Shaoxing, and Jinhua increased from the “low” to the “general” type, while the rest of the cities still belonged to the “low” category. Generally, there are three core spatial distribution patterns: those of Hangzhou, Shanghai, and Nanjing. The innovation output capacity of most prefectures and cities has obviously improved; the number of innovation outputs has increased, and differentiation has been enhanced.

(3) The innovation growth pattern of the YRD based on innovation input and innovation output (Figure 4). In this study, the Getis-OrdGi* index of the spatial statistical analysis module in the ArcGIS software is used to characterize and analyze the changes in the growth patterns of the hotspots of innovation. The results show the presence of a few innovation growth hotspots in the YRD between 2004 and 2019 based on science and technology expenditure and patent number; the former only includes Shanghai, while the latter includes Nanjing, Suzhou, Shanghai, and Hangzhou. The number of sub-hot areas of science and technology expenditure is higher and more concentrated than that of the hotspot areas, whereas the number of areas for patent licensing is several times fewer and its distribution is scattered. Most cities in the YRD comprise cold or sub-cold areas.

The increase in Shanghai’s innovation investment based on science and technology expenditure accounts for 27.08% of the total increase in innovation investment in the YRD. Based on the number of patents granted, the increments in the innovation output of the cities of Nanjing, Suzhou, Shanghai, and Hangzhou account for 9.59%, 11.72%, 15.06%, and 9.62%, respectively, of the total increase in the innovation output of the YRD, which is 45.99% in total. This is basically attributed to the aforementioned mechanism of the spatial agglomeration hotspot of the growth of innovation in the YRD based on science and technology expenditure and patent number. Based on the growth of innovation investment (calculated using scientific and technological expenditures) in the YRD between 2004 and 2019, the proportion of hotspots, secondary hotspots, secondary cold spots, and cold spots were calculated as 4%, 16%, 32%, and 48%, respectively. The hotspots of innovation investment growth show a spatial distribution with Shanghai as the single core, while the sub-hot and sub-cold areas are mainly distributed in a “Z” type of distribution belt centered on Shanghai hotspots. This shows that the cities in the sub-hot and sub-cold spots are influenced by the space radiation of the cities in the hotspots and have positive interactions. The sub-cold zone is also sporadically distributed, such as Xuzhou in the north of Jiangsu and Taizhou in the southeast of Zhejiang. The cold regions are agglomerated and are mainly distributed in the central and northern part of Jiangsu and the western and southern parts of Zhejiang, while the cold spots indicate that the development potential of innovation investment agglomeration based on science and technology expenditure is low. Based on the growth of innovation output (which was calculated using the number of patents granted) in the YRD region between 2004 and 2019, the proportion of hotspots, secondary hotspots, secondary cold spots, and cold spots were calculated to be 16%, 12%, 48%, and 24%, respectively. The spatial distribution of the hot areas of innovation output growth in the YRD has three core distributions: Shanghai and Suzhou, Nanjing, and Hangzhou; additionally, the radiation ability of Shanghai was enhanced. There are only three sub-hotspots: Wuxi, Ningbo, and Wenzhou. The distribution of the sub-cold zone is relatively concentrated, mainly around the hot zone. The number of cold regions is small, and they are clustered, mainly distributed in Lianyungang, Suqian, and Huaian in northern Jiangsu, and in Quzhou, Lishui, and Zhoushan in southwestern Zhejiang.

From 2004 to 2019, the growth of innovation output in the YRD based on the number of patents granted in the hotspot area and the sub-cold spot area increased compared to the growth of innovation input based on science and technology expenditure. This indicates that an increasing number of cities in the YRD pay more attention to the important role of innovation in regional development, which is consistent with the regional integration development strategy and innovation-driven development strategy of the Changjiang Delta. At the same time, Shanghai is a hotspot in terms of innovation input and output, which shows high innovation investment and output activity in Shanghai and a significant high concentration pattern. Shanghai plays a key role in driving innovation in neighboring cities, which is closely associated with Shanghai’s superior geographical location, highly developed economy, and national policy support. Lianyungang, Suqian, and Huaian in Jiangsu Province along with Quzhou, Lishui, and Zhoushan in Zhejiang Province are categorized as cold spots in terms of innovation input and output. The level of urban economic development in these peripheral regions has been observed to be relatively low, and the degree of innovation input and output concentration is weak. Therefore, there is still a lot of room for improvement in the future.

### 3.2. Spatial Pattern Analysis of Water Pollution

Water pollution is characterized by the discharge of industrial wastewater, and classification and mapping are carried out by the natural breaking point classification method in the ArcGIS software. From the perspective of the spatial evolution pattern, the water pollution agglomeration pattern in the YRD has evolved from the original “Z” pattern—represented by Shanghai, Suzhou, Wuxi, Changzhou, Zhenjiang, Nanjing, Jiaxing, Hangzhou, Shaoxing, and Ningbo—to a new agglomeration pattern, with Shanghai and Suzhou as the core.

**Figure 5:**
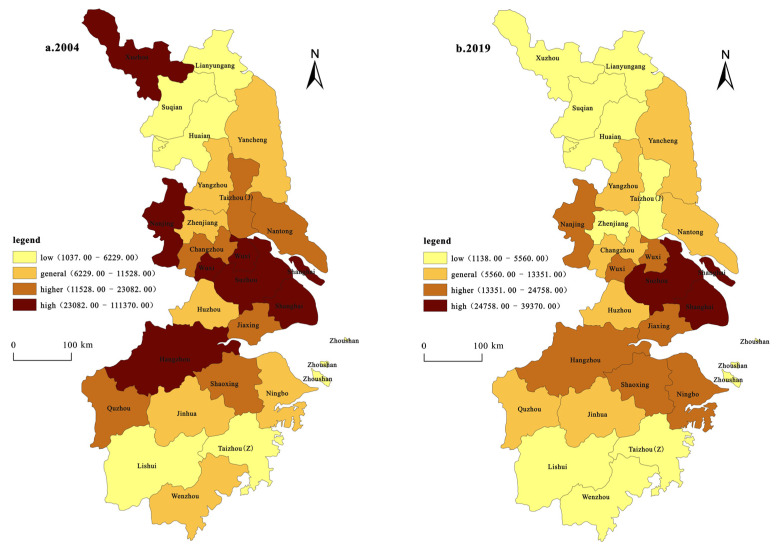
Changes in the spatial pattern of water pollution in the YRD in 2004 and 2019.

In terms of the spatial distribution of water pollution, the pollution is highly concentrated in Shanghai, Suzhou, and its surrounding areas (Figure 5). In 2004, six cities were “high” pollution types, including Hangzhou, Shanghai, Suzhou, Wuxi, Nanjing, and Xuzhou, accounting for 24% of the total pollution. The “higher” pollution-type areas were Quzhou, Shaoxing, Jiaxing, Changzhou, Nantong, and Taizhou, accounting for 24%. The “general” pollution-type areas were Wenzhou, Jinhua, Ningbo, Huzhou, and Zhenjiang, accounting for 28%. The rest of the cities were “low” pollution areas, accounting for 24%. Overall, the distribution of cities with various pollution types was relatively scattered, and most of the cities with general pollution types and above were concentrated in the middle of the YRD. Compared with 2004, the only cities with “high” pollution types in 2019 were Suzhou and Shanghai, with “high” pollution types accounting for 8%. Hangzhou, Shaoxing, Ningbo, Jiaxing, Wuxi, and Nanjing were the “higher” pollution-type cities, accounting for 24%; the cities with a “general” pollution type were Quzhou, Jinhua, Huzhou, Changzhou, Yangzhou, Yancheng, and Nantong, accounting for 28%. The remaining 10 prefecture-level cities were “low” pollution areas, accounting for 40%.

In terms of the time evolution pattern, since 2004, the environmental pollution in most regions of the YRD has shown a downward trend. Hangzhou, Nanjing, and Wuxi changed from “high” pollution-type cities to “higher” pollution-type cities, whereas Quzhou, Changzhou, and Nantong changed from “higher” pollution-type cities to “general” pollution-type cities. The number of “low” pollution-type cities increased by four; namely, Xuzhou has changed from a “high” pollution-type to a “low” pollution-type city, Taizhou has changed from a “higher” pollution city to a “low” pollution city, and Zhenjiang and Wenzhou have changed from a “general” pollution city to a “low” pollution city. This shows that with the increasingly stringent environmental regulations and the enhancement of environmental protection awareness, most of the YRD region has increased investment in the reduction of water pollution. As a result, the water pollution situation has begun to ease, and the quality of economic development has been increasingly enhanced.

## 4. Analysis of the Spatial Coupling between the Center of Gravity of Technological Innovation and the Center of Gravity of Environmental Pollution in the YRD

### 4.1. Characteristics of the Migration Trajectory of the Center of Gravity of Technological Innovation and That of Water Pollution in the YRD

The trend of changes in the center of gravity of innovation output and innovation input in the YRD is basically the same and has been moving along the northwest direction in Jiaxing, Suzhou and Wuxi, especially before 2015. The center of gravity of water pollution changed along the northeast direction near Changzhou, Huzhou, and Suzhou, indicating that the impact of technological innovation on water pollution was not significant. This is because most of the innovation output and innovation input are designed to focus on industrial production, and that there are fewer energy-saving and environmental protection technologies. As environmental regulations have been increasingly reinforced, the centers of gravity of innovation output and innovation input moved to the southwest after 2015, whereas the center of water pollution moved to the southeast, and the effect of technological innovation on water pollution began to appear to a certain extent.

**Figure 6:**
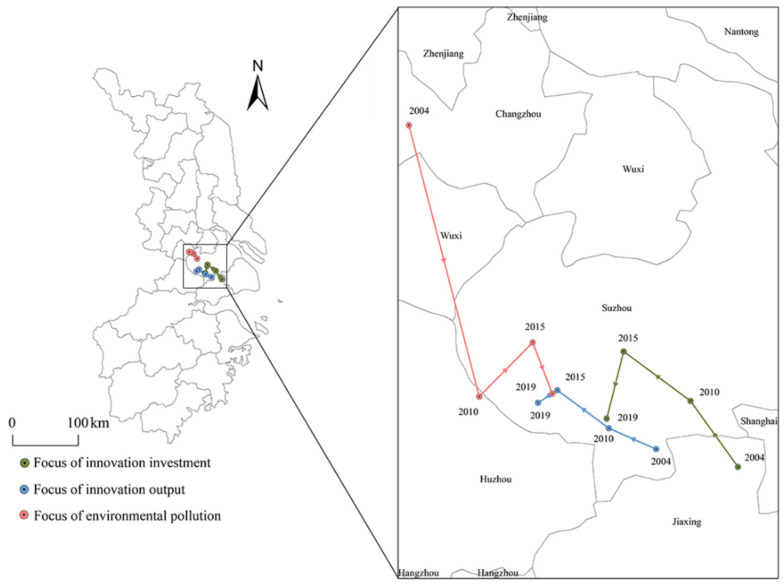
The migration trajectory of the center of technological innovation and the center of water pollution in the Yangtze River Delta.

More specifically, the centers of gravity of innovation output, innovation input, and water pollution in the YRD were in Jiaxing-Suzhou-Suzhou-Suzhou, Suzhou-Suzhou-Suzhou-Suzhou, and Changzhou-Huzhou-Suzhou-Suzhou, but before 2015, the focus of innovation output, innovation input, and the change trajectory of water pollution were basically the opposite. After 2015, the trajectory of the center of gravity of the three began to have a certain similarity (Figure 6).

### 4.2. Coupling between the Center of Gravity of Technological Innovation and That of Water Pollution

**Figure 7:**
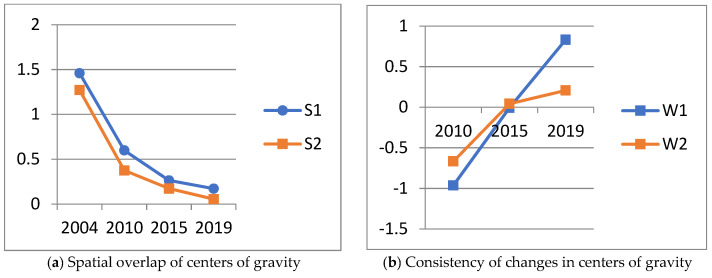
Changes in the spatial overlap and consistency of change indices of technological innovation and environmental pollution in the YRD.

From the perspective of spatial overlap, whether it is the center of patent innovation output or innovation input, its spatial overlap with the center of gravity of water pollution shows a downward trend as a whole (Figure 7). The spatial overlap between the center of gravity of both types of innovation and the center of gravity of water pollution basically remained the same. However, the spatial overlap between the centers of gravity of innovation input and water pollution fluctuated relatively more, with the distance between the two centers of gravity remaining at approximately 0.17–1.46. In contrast, the distance between the center of gravity of patent-based innovation output and that of water pollution was relatively smaller, with the distance between the two centers of gravity ranging from approximately 0.05 to 1.27. The fluctuation was smaller, and the change was relatively stable. This shows that the spatial coupling correlation between the center of gravity of patent-based innovation output and that of water pollution is slightly stronger and smoother than that based on innovation inputs.

From the perspective of the consistency of changes, the innovation center of patent output and innovation input along with the consistency of changes in water pollution both show an upward trend. More specifically, the two types of innovation centers of gravity and water pollution centers of gravity tend to be the same overall. Before 2015, the consistency between the center of innovation output based on patents and the center of environmental pollution was relatively high. In 2019, the consistency between the center of gravity of innovation input and the center of environmental pollution was relatively high. This is consistent with the above conclusion on the spatial overlap of the centroids.

When spatial overlap and consistency were combined, the coupling between the centers of gravity of innovation and pollution in the YRD was weak, although it appeared to be increasing. Its spatial overlap showed a downward trend, with a larger decline before 2015 and a weakened decline thereafter; the consistency of changes continued to increase, indicating that the directions of change for the two centers of gravity gradually evolved from inconsistent to relatively consistent.

Note: S1 represents the spatial overlap between the centers of gravity of input-based innovation and patent-based innovation output, and S2 represents that of environmental pollution. W1 and W2 represent the consistency of changes in the center of gravity of these two types of innovation and the center of gravity of environmental pollution, respectively. The vertical axis of Figure 7a,b shows the consistency of the spatial overlap and change between the two types of technological innovation and the water pollution center of gravity, respectively. The horizontal axes of Figure 7a,b are both years.

## 5. Effects of Technological Innovation on Water Pollution in the YRD

### 5.1. Selection of Influencing Factors

Based on the basic data on innovation output, innovation input, and water pollution in the YRD from 2004 to 2019, this study attempts to quantitatively measure the impact of technological innovation on water pollution using the gray correlation analysis method. After comprehensive consideration of the main factors influencing water pollution, eight aspects were selected: industrial structure, innovation environment, urban greening, environmental governance, population size, FDI, innovation input, and innovation output, which presented nine independent variables. This study assumes that innovation input and patent innovation output have certain effects on water pollution in addition to seven control variables (Table 1). Industrial structure may have a certain impact on the water, and the acceleration of industrialization processes is often accompanied by excessive development and the pollution emissions of resources. Technological progress in the wake of the development of the social economy will optimize and upgrade the industrial structure to a certain extent, and the decline of the proportion of the secondary industry in the national economy will ease the pressure on resources and the environment. More specifically, the proportion of the secondary industry in GDP is selected to measure the industrial structure. According to past studies, basic innovation has a significant positive and direct impact on scientific and technological innovation, and communication plays an important role in the basic function of innovation. The year-end number of mobile phone users and the postal service income were selected as representatives. Urban greening can reflect the degree of greenness of a city, the degree of attention to environmental protection, and the specific selection of green coverage rates to measure greenness. Pollution governance has a significant impact on water pollution; therefore, general industrial solid waste is used as a proxy variable of pollution control. The change in population affects the environmental carrying capacity, which directly affects resources and the environment. Population size refers to the total population at the end of the year. FDI tends to cause pollution input and transfer; therefore, the actual use of foreign capital in the year was selected to represent the FDI factors. Numerous factors influence technological innovation, but the most significant ones are innovation input and innovation output. Therefore, science and technology expenditure is used to represent innovation input, and the number of patent licenses is used to represent innovation output.

### 5.2. Gray Correlation Analysis

The gray correlation degree was used to explore the impact of technological innovation on water pollution in the YRD from 2004 to 2019 (Table 2). The results showed that industrial structure and water pollution had the highest gray correlation degree (0.5289), indicating that the industrial structure has the greatest impact on water pollution, and that the secondary industry is an important source of environmental pollution. Industrial upgrading and transformation, the proportion of secondary industry, and development quality have a significant impact on the environmental conditions. Furthermore, industrial structure is an important factor affecting regional environmental quality and has an obvious pulling effect on pollutant discharge [4].

The gray correlation degree between environmental governance factors and water pollution was 0.4602, with environmental governance factors also having a significant impact on water pollution. The intensity and utilization efficiency of environmental governance will directly affect the discharge of pollutants and effectively improve the environmental situation. Furthermore, the gray correlation degree between population size and water pollution was 0.4591, indicating that population size has significant impact on water pollution.

Meanwhile, the gray correlation degrees of urban greening with FDI and water pollution were 0.4568 and 0.336, respectively, and its impact on water pollution was intermediate. Urban greenness can improve the environment to a certain extent, and the greening rate is an indication of the emphasis and attention given by government departments to environmental quality. The FDI has a moderate effect on water pollution. This shows that although FDI input and foreign industrial transfer increase the pollution concentration degree of domestic regions, the impact of FDI on water pollution is continuously attenuated by the increasing implementation of stringent environmental policies, targeted industrial transfer, and foreign investment.

The postal service revenue, which is the basis for innovation, had a lower correlation with water pollution (0.2546). Consequently, and given the continuous popularization of phones and the Internet following societal and communication development, the significance of postal services with respect to innovation continues to decline; therefore, it has a lower impact on water pollution.

The gray correlation degree of mobile phone users and water pollution was 0.1863, which had the least impact on water pollution. Relevant studies found that the development of the digital economy significantly reduced the emissions of various environmental pollutants in cities, while green innovation and industrial structure optimization were important mechanisms for the digital economy to reduce the emissions of environmental pollutants in cities [30]; technological progress and structural upgrading were key transmission paths for the Internet to affect environmental quality [41]. However, traditional industries still account for a relatively large proportion.

Finally, the correlations between the innovation input and innovation output to water pollution were low: 0.2635 and 0.281, respectively. Advanced technological innovation is based on the pursuit of efficiency, not the pollution of the environment. Continuous economic development requires the consumption of a lot of scarce non-renewable resources and technological innovation to increase productivity. New sources of pollution may therefore be formed, In this case, environmental improvement is temporal owing to technological restrictions [5].

**Figure 8:**
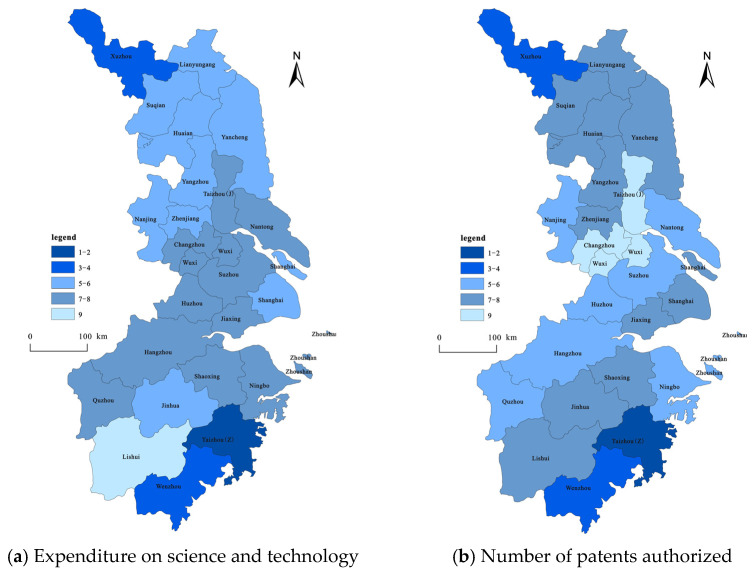
Spatial pattern of gray correlation degree ranking between technological innovation and water pollution in the YRD.

Based on the above analysis, the gray correlation analysis method was used for each city in the YRD to further analyze and compare the spatial pattern of technological innovation and the water pollution gray correlation ranking from 2004 to 2019. The results showed that the number of cities in which the gray correlation between innovation input, innovation output, and water pollution is at the medium-to-higher levels is approximately the same (Figure 8).

More specifically, in most cities, the correlation between innovation input and water pollution is low, mainly concentrated in the central region of the YRD. Innovation output was also less associated with water pollution in most cities, which were mainly concentrated in the northern and southern regions of the YRD. This further confirms that both innovation output and innovation input have less impact on water pollution. Therefore, these regions need to focus on optimizing the industrial structure and increasing investment in green innovation while increasing investment in research and development.

Note: The numbers in the legend of Figure 7a are the rankings of the role of innovation input in influencing the factors of water pollution for each city; the numbers in the legend of Figure 7b are the rankings of the role of innovation output in influencing the factors of water pollution in each city.

## 6. Discussion

The center of patent innovation output and the center of gravity of innovation input are essentially the same, but the centers of gravity of both are the opposite of the center of gravity of water pollution. A quantitative analysis of the gray correlation model also confirms that both patent innovation output and innovation input are not related to water pollution, indicating that China’s environmental conditions are more influenced by industrialization; the impact of technological innovation on water pollution also remains relatively weak. Other studies confirm this view: in the process of economic development, technological innovations focus on profitability in the industrial production process and ignore technological innovation for environmental protection; for example, there is a lack of green patents in the industry. Green innovation can significantly improve environmental sustainability only under an environmental regulation policy, and technological innovation will promote the mitigation of environmental pollution [5,41]. Therefore, to effectively improve the water situation, it is important to formulate a sound environmental policy, reduce environmental pollution emissions, encourage enterprises to increase green technology innovation research and development, and promote technological innovation for the amelioration of environmental pollution. All localities need to improve their informatization level, optimize their industrial structure, promote industrial upgrading, especially improve the green technology content of the industrial production process, and take the digital revolution as the starting point for the cultivation of new industries. Next, they need to further strengthen environmental governance, increase environmental governance and investment, improve the ecological environment while ensuring rapid economic development, implement a series of policies and measures to strengthen the pollution discharge in the production process, and reasonably control the population size. Finally, they need to intensify afforestation, improve the green coverage rate of built-up areas, and mitigate environmental pollution.

## 7. Conclusions

Using ArcGIS, DPS, CorelDraw, and other software, this study analyzed the spatial pattern of technological innovation in the YRD from 2004 to 2019 and its impact on environmental pollution. The results showed that:

(1) The spatial pattern of innovation investment in the YRD has an obvious single-center structure that tends to spread. The spatial pattern based on innovation output is quite different, and the diffusion effect is relatively more obvious. Most cities’ innovation output capacity has obviously improved, the number interval of innovation output has increased, and the differentiation has been strengthened. From a single spatial pattern, with Shanghai as the core, it has evolved into a situation with three core structures: Hangzhou, Shanghai, and Nanjing.
(2) A few innovation growth hotspots are based on innovation input and output, among which the former only includes Shanghai, while the latter includes Nanjing, Shanghai, and other regions. Compared with hotspots, the number of innovation input sub-hotspots is high and relatively concentrated, whereas the number of sub-hot zones of patent innovation output is less than that of hotspots; the distribution is also relatively scattered. Hence, most cities are covered by cold-point areas or sub-cold areas.
(3) The agglomeration pattern of water pollution in the YRD has evolved from the initial “Z” pattern—represented by Shanghai, Suzhou, Wuxi, Changzhou, Zhenjiang, Nanjing, Jiaxing, Hangzhou, Shaoxing, and Ningbo—to a new pattern of stripes, represented by Shanghai and Suzhou. The overall situation of water pollution is constantly improving.
(4) The center of gravity of patent innovation output in the YRD is seen to basically follow the same trend of change as the innovation input center of gravity, which shifts in Jiaxing, Suzhou, and Wuxi in a northwest direction, especially before 2015; on the other hand, the center of gravity of water pollution evolved in the opposite direction, changing in the southeast direction. Whether it is the center of patent innovation output or the center of innovation input, its spatial overlap with the center of gravity of water pollution shows an overall downward trend, and the consistency of the two with the change in water pollution shows an upward trend.
(5) The gray correlations of industrial structure, environmental governance, and population size to water pollution are high, followed by FDI and the urban greening rate; in contrast, that of technological innovation to water pollution is relatively low, which is mainly related to the type and goal of innovation. The proportion of cities with innovation inputs, innovation outputs, and water pollution gray correlation at the medium-to-high level is approximately the same, and most cities have a low water pollution gray correlation.

## Figures and Tables

**Table 1:** Factors influencing location selection of water pollution in the YRD.

Influencing Factors	Representative Indicators (Unit)
Industrial structure	Share of secondary sector in GDP (%)
Foundations of innovation	Year-end mobile phone subscribers (RMB million)/Revenue from postal services (RMB million)
Urban greenery	Greenery coverage in built-up areas (%)
Environmental Governance	General Industrial Solid Waste Integrated Utilization Rate (%)
Population size	Total population at the end of the year (10,000)
FDI Factor	Actual amount of foreign investment used in the year (USD million)
Investment in innovation	Expenditure on science and technology (RMB million)
Innovation output	Number of patents granted (pieces)

**Table 2:** The gray correlation between water pollution in the Yangtze River Delta and various factors from 2004 to 2019.

Influencing Factors	Gray Correlation	Sequence
Share of secondary sector in GDP	0.5289	1
General Industrial Solid Waste Integrated Utilization Rate	0.4602	2
Total population at the end of the year	0.4591	3
Greenery coverage in built-up areas	0.4568	4
Actual amount of foreign investment used in the year	0.336	5
Number of patents granted	0.281	6
Expenditure on science and technology	0.2635	7
Revenue from postal services	0.2546	8
Year-end mobile phone subscribers	0.1863	9

## Data Availability

The data presented in this study are available on request from the corresponding author.
